# Supplementary material for: Exploring the prevalence of severe obesity in a large teaching hospital
Source: PLoS One. 2025 Sep 19;20(9):e0332608. doi: 10.1371/journal.pone.0332608 (PMC12448348; doi:10.1371/journal.pone.0332608)
Supplement: Attachment — Submitted filename: Response to Reviewers.docx [file pone.0332608.s004.docx]

Dear Editor and Reviewers,

We sincerely thank you for the careful evaluation of our manuscript and for the constructive and insightful comments provided. We have addressed each point in detail below. All changes have been incorporated into the revised manuscript, with tracked changes enabled. We believe these revisions have improved the clarity and scientific value of our work.

Reviewer #1

Comment 1:
“The Lancet Commission on the Definition and Diagnosis of Clinical Obesity recommends defining and diagnosing clinical obesity based on the presence of 'excess or dysfunctional adiposity that impairs health,' irrespective of body mass index. I suggest incorporating this definition into both the Introduction and Discussion sections of your manuscript, as it may influence the accurate estimation of obesity prevalence in your study population.”

Response:
Thank you for this important suggestion. We have incorporated the recommended definition in both the Introduction and Discussion sections. Specifically, we now refer to the Lancet Commission on Clinical Obesity and discuss how this broader definition highlights the limitations of relying solely on BMI for obesity classification.

Comment 2:
“An important subgroup worth considering is patients with cancer, particularly those with obesity-related cancers. Including this subgroup in your analysis could provide valuable insights and strengthen your findings. Would it be possible to add this population to your study cohort?”

Response:
We appreciate this thoughtful suggestion. We agree that the intersection of obesity and cancer is an important area for future research. However, within the current scope and dataset of this retrospective study, cancer-specific subgroup analyses could not be reliably performed due to variability in diagnostic coding and low prevalence per specific cancer subtype. We have acknowledged this limitation in the revised Discussion and suggested it as a valuable direction for

Reviewer #2

Comment 1:
“Please change 'bariatric surgery' to 'Metabolic Bariatric Surgery (MBS)' throughout the manuscript, in line with IFSO recommendations.”

Response:
Thank you for pointing this out. We have replaced the term “bariatric surgery” with “Metabolic Bariatric Surgery (MBS)” at all indicated locations:
→ Line 36, 120, 314, 320
We have also updated the reference title accordingly if the original title allowed it; if not, we retained the original title as per citation integrity guidelines and clarified the terminology in our manuscript body.

Comment 2:
“Please change ‘comorbidities’ to ‘obesity-associated diseases/disorders’.”

Response:
We have updated the terminology where appropriate to “obesity-associated diseases,” particularly in line 415. Other instances were reviewed and deemed contextually appropriate or already aligned with the suggested terminology.

Optional Comment:
“Add IFSO-aligned BMI classification (Obesity class II–V).”

Response:
Thank you for this suggestion. While we recognize the value of the IFSO classification system, we chose to maintain the definition of severe obesity as BMI ≥35 kg/m² for clarity and consistency with national statistics used in this study (CBS, RIVM). Moreover, we did not include the IFSO subcategories (Obesity class II–V) as a subgroup analysis due to small sample sizes in the higher BMI classes, which would limit statistical reliability. We now acknowledge the IFSO classification system in the revised Discussion as a potential framework for future stratified analyses when larger datasets are available.

Once again, we are grateful for the reviewers' thoughtful feedback and believe the revised manuscript has improved significantly as a result. Please do not hesitate to contact us for further clarification or revisions.

With kind regards,
Ephrahim E Jerry, on behalf of all co-authors
